# Acute Right Ventricular Failure Immediately Following Subxiphoid Pericardial Window Requiring Temporary Cardiopulmonary Bypass Support: A Case Report of Pericardial Decompression Syndrome

**DOI:** 10.7759/cureus.26286

**Published:** 2022-06-24

**Authors:** Ashie Kapoor, Yi McWhorter, Dean R Polce

**Affiliations:** 1 Anesthesiology, MountainView Hospital, Las Vegas, USA; 2 Anaesthesiology, MountainView Hospital, Las Vegas, USA

**Keywords:** paradoxical hemodynamic collapse, pericardial decompression syndrome, right ventricular failure, pericardial window, cardiac tamponade

## Abstract

Cardiac tamponade is a life-threatening condition requiring emergent intervention, such as a pericardial window, to drain the pericardial effusion, subsequently improving cardiac output. A rare complication of this procedure is pericardial decompression syndrome which results in a paradoxical cardiovascular collapse. A 65-year-old male with bacterial endocarditis status posts mitral and aortic valve replacement presented for an emergent subxiphoid pericardial window to relieve cardiac tamponade. After draining 850mL of pericardial fluid, the patient suffered a cardiac arrest secondary to acute right ventricular failure seen on an intraoperative transesophageal echocardiogram (TEE). Despite manual compressions and high-dose vasopressors, hemodynamics did not improve, and the patient was emergently placed on cardiopulmonary bypass (CPB) support. Within two hours, CPB support was successfully weaned. Temporary CPB can improve acute right ventricular failure following pericardial decompression without needing longer-term extracorporeal support.

## Introduction

Pericardial effusion can develop following routine cardiac surgeries. Generally, these effusions are small and inconsequential. However, sometimes they can persist past the immediate postoperative phase, progress in size, and even cause tamponade physiology. Cardiac tamponade carries a high mortality risk and must be addressed promptly. A rare complication of this procedure is pericardial decompression syndrome (PDS) which results in paradoxical cardiovascular collapse. This is usually due to left ventricular failure, or rarely, right ventricular failure. Although rare, this syndrome carries a 30% mortality risk and thus requires vigilance when caring for these patients [1]. We present a patient with acute right ventricular failure following surgical drainage of pericardial tamponade, requiring immediate institution of full cardiopulmonary bypass support until sufficient recovery of his right ventricular function.

## Case presentation

A 65-year-old male with an extensive past medical history presented from an outside hospital for evaluation of bacterial endocarditis and new-onset right-sided weakness concerning an embolic cerebrovascular accident. The patient had a history of severe aortic stenosis, which was treated with transcatheter aortic valve replacement one year prior to presentation. He also had a history of myocardial infarction status post percutaneous coronary intervention, poorly controlled diabetes mellitus, chronic kidney disease stage 3, and peripheral vascular disease requiring left common femoral endarterectomy complicated by multiple wound infections. On admission, a transesophageal echocardiogram (TEE) showed small mobile vegetation on the mitral valve with severe mitral regurgitation. The left ventricular ejection fraction was noted to be 60-65%. Right ventricle systolic pressure (RVSP) was measured as 60mmHg using the Doppler method, though the systolic function was normal. The patient was started on antibiotics for enterococcal bacteremia and scheduled for mitral valve replacement. Intraoperatively, second vegetation was also found on the bioprosthetic aortic valve and thus was replaced. The patient tolerated the procedure well and required minimal vasoactive medications. He continued to depend on epicardial pacing and was scheduled for permanent pacemaker placement after completing antibiotics for bacteremia. The patient’s postoperative course was complicated by worsening kidney function, ultimately requiring hemodialysis.

On postoperative day 6, the patient suffered a cardiac arrest ten minutes after the initiation of hemodialysis. He was reintubated, and spontaneous circulation (ROSC) was returned after 8 minutes of cardiopulmonary resuscitation. The etiology of the cardiac arrest was not immediately clear. A transthoracic echocardiogram (TTE) was performed immediately and showed a trivial pericardial effusion with mildly reduced right ventricular systolic function. The patient rapidly improved and was able to be extubated the next day. However, hemodialysis was attempted one day later, during which the patient again suffered cardiac arrest. ROSC was achieved after 5 minutes of cardiopulmonary resuscitation and cessation of hemodialysis. A TTE was performed at the bedside a few hours after this event. The patient was found to have a large circumferential pericardial effusion with features concerning tamponade physiology. He was then emergently taken to the operating room for a subxiphoid pericardial window.

**Figure 1:**
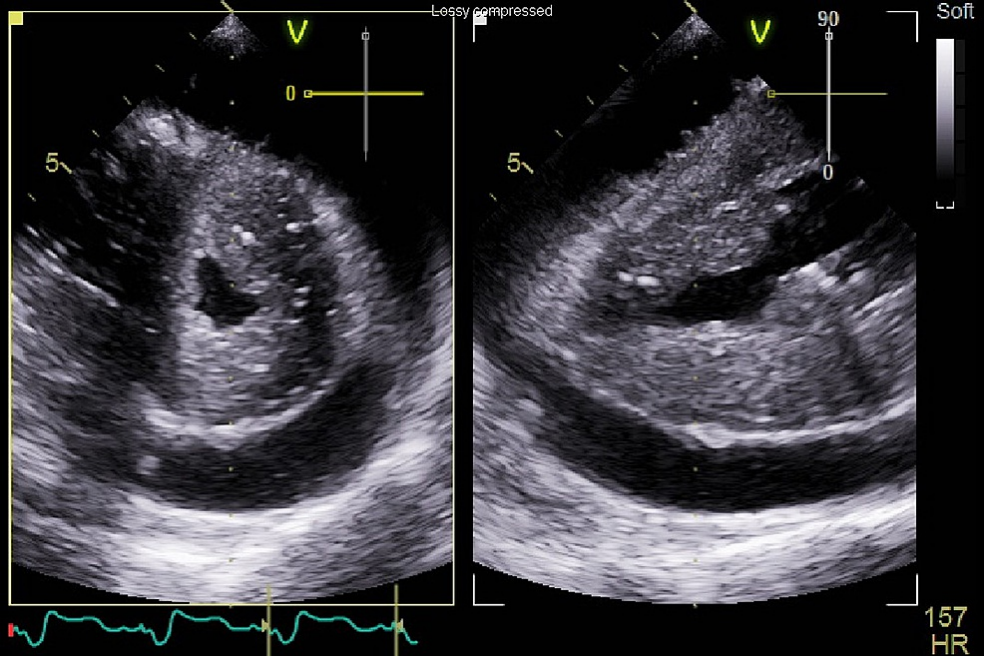
Preoperative transesophageal echocardiogram shows large circumferential pericardial effusion in transgastric short axis (left) and long-axis (right) views.

**Figure 2:**
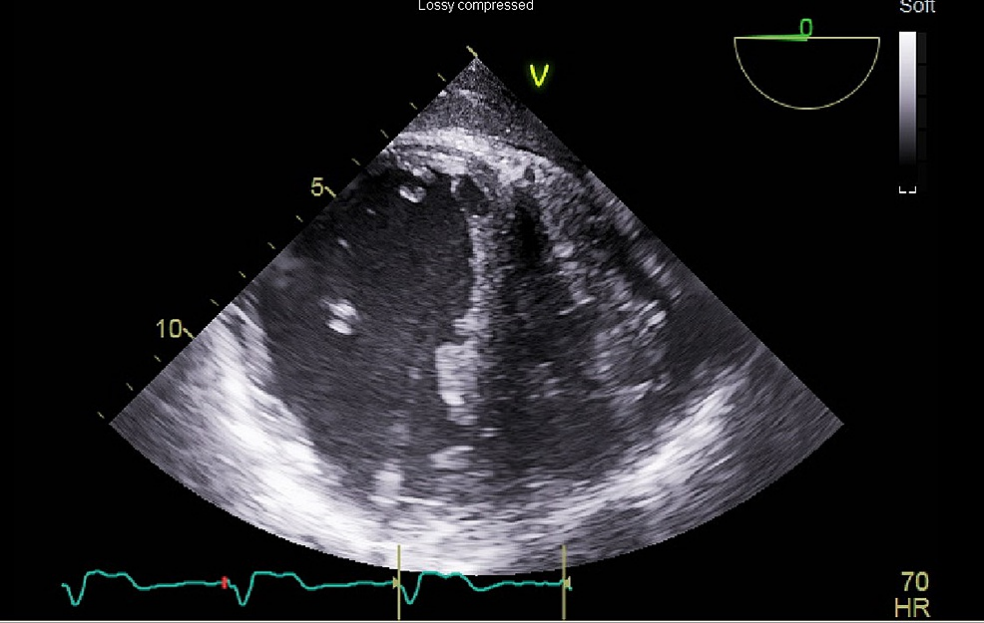
Transesophageal echocardiogram transgastric short-axis view shows severe right ventricular dilation immediately following surgical pericardial drainage.

The patient was brought to the operating room, and inhalational induction with sevoflurane was done through an existing endotracheal tube. Figure 1 shows intraoperative TEE immediately prior to pericardial effusion drainage. Our surgeon was able to drain 850mL of pericardial fluid. However, unexpected hemodynamic deterioration ensued. Intraoperative TEE showed a severely dilated, hypokinetic right ventricle and D-shaped septum, consistent with acute right ventricular failure. Pulseless electrical activity cardiac arrest occurred, and chest compressions were started. Given the lack of ROSC or right ventricular recovery, the surgeon converted to sternotomy and continued manual cardiac compressions. When the chest was opened, the patient had partially recovered, but the right ventricle remained severely dilated and hypokinetic (Figure 2). He was emergently placed on cardiopulmonary bypass (CPB) for temporary extracorporeal support and decompression of the right ventricle.

**Figure 3:**
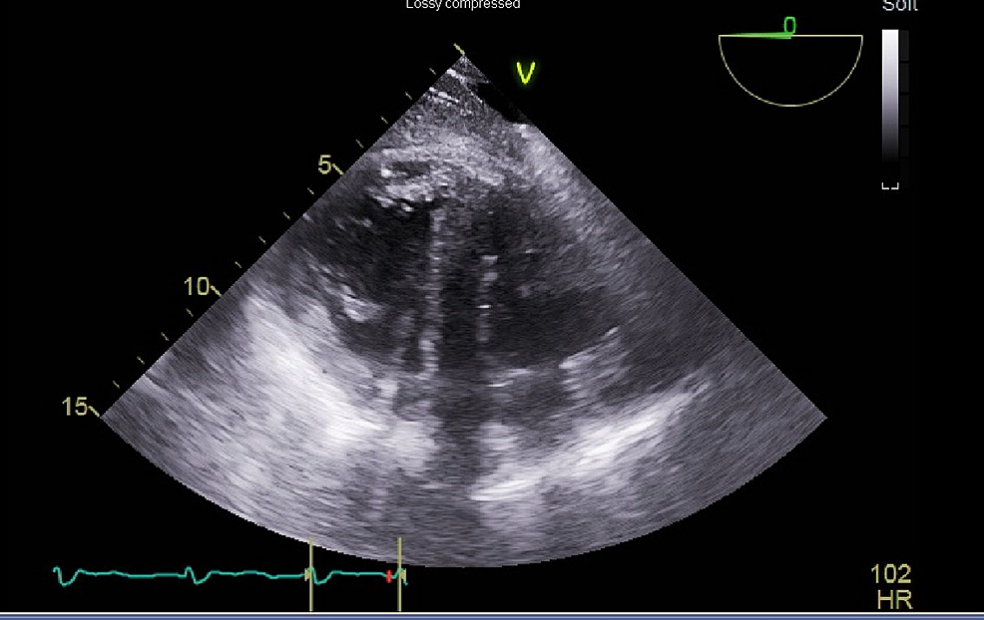
Transesophageal echocardiogram transgastric short-axis view shows improved right ventricular size after separation from the cardiopulmonary bypass machine.

The first attempt to wean from CPB after an hour was unsuccessful. The patient remained on bypass for another half an hour, and an additional liter of fluid was removed. A second attempt at weaning from bypass was successful. However, the patient required significant inotropic and vasopressor support, including epinephrine infusions at 10mcg/min, dobutamine at 10mcg/kg/min, milrinone at 0.5 mcg/kg/min, and norepinephrine at 10mcg/min. After hemostasis was achieved and the sternum was closed, TEE showed right ventricle function quite improved and less dilated, though still hypokinetic (Figure 3). The left ventricle was noted to be functioning normally. The interventricular septum was positioned more midline and ventricular interdependence improved. The patient was transported to the intensive care unit intubated and on four inotropic and vasopressor infusions, which were weaned down slowly over the next few days. TTE performed four days after surgery showed normal left ventricular and right ventricular size and function with an ejection fraction of 50-55%. The patient continued to recover in the intensive care unit and was later extubated successfully, transferred to the floor, and eventually discharged from the hospital.

## Discussion

Pericardial effusions can occur after cardiac surgeries, and most are asymptomatic and resolve by postoperative day 10. Up to 6% persist and develop into late cardiac tamponade, requiring invasive treatment. Most of these pericardial effusions occur following a single-valve surgery instead of multiple-valve or coronary artery bypass grafting procedures [2]. Treating pericardial effusion requires drainage via either needle pericardiocentesis or a surgical pericardial window. In the case of obstructive shock due to pericardial tamponade, this allows a hemodynamic profile to improve. However, rare paradoxical cardiovascular collapse can happen the following drainage. This is known as pericardial decompression syndrome, previously referred to as low cardiac output syndrome. The incidence of this syndrome has been reported as 4.8-11% of patients with pericardial effusion, though the true incidence remains unknown due to selection bias and reporting discrepancies [3]. It is present immediately after drainage of pericardial effusion, up to 48 hours afterward. Pradhan et al. reported that the most common presentation of PDS is left ventricular failure, and the least common is right ventricular failure. They also reported a mortality rate of 30% associated with surgical drainage [1,4]. Thus, prevention of PDS includes alternatives to surgical pericardial window such as drainage via needle pericardiocentesis or surgical drainage of only enough fluid to resolve tamponade followed by slow drainage via an indwelling drainage catheter [3]. These alternatives have not been studied, and thus efficacy of decreasing PDS with these methods is unknown.

The etiology of PDS is complex, with several proposed theories. The hemodynamic hypothesis states that with acute drainage of pericardial fluid, venous return to the right heart is increased, resulting in rapid right ventricular expansion and sometimes leading to failure. Right ventricular failure results in left ventricular failure via decreased forward flow to left ventricular end-diastolic volume and septum shifts towards the left ventricle, further decreasing preload [3-5]. We believe our patient’s two cardiac arrests prior to pericardial window surgery are unlikely related to or predictive of his PDS following effusion drainage. These arrest mechanisms are thought to be due to preload reduction with hemodialysis initiation. Following decompression of his pericardial effusion, our patient’s hemodynamic status was expected to improve. However, severe right ventricular dilation and circulatory collapse ensued, consistent with a proposed hemodynamic mechanism of PDS. After temporary support with CPB, his second attempt of weaning was successful likely due to decreased preload and ventricular wall stress after removing one liter of fluid and further escalation of inotropic support of the right ventricle.

Other theories for PDS include the ischemic hypothesis and the autonomic imbalance hypothesis [3,4]. The ischemic hypothesis refers to decreased coronary perfusion secondary to high pericardial pressures resulting in stunned myocardium. Our patient had normal coronary blood flow, to begin with, and it is unlikely that ischemia was the underlying cause of PDS. However, severe systemic hypotension during right ventricular failure could have exacerbated further hemodynamic compromise. The autonomic imbalance hypothesis refers to a decrease in sympathetic stimuli after the resolution of tamponade. This could explain why inotropic agents such as epinephrine are crucial to preventing further systolic dysfunction in this syndrome.

To the best of our knowledge, this is the first case of pericardial decompression syndrome causing acute right ventricular failure managed with emergent cardiopulmonary bypass. Liao et al. reported a similar case in which the patient required emergent veno-arterial extracorporeal membrane oxygenation (VA-ECMO) to maintain hemodynamic support and was weaned off by postoperative day 7 [6]. In the current case, intraoperative discussions were held regarding VA-ECMO; however, given the quicker availability of the CPB machine, the surgical team opted for that. There were also considerations made to right ventricular assist devices. However, our patient could sustain native circulation after two hours of CPB and significant inotropic and vasopressor support. As reported previously, the time to recovery following PDS is variable, with some patients recovering quickly with pharmacologic support and others requiring more invasive management. Our patient recovered in the intensive care unit, weaning off inotropic and vasopressor support. He was eventually extubated and transferred to the floor.

## Conclusions

Pericardial decompression syndrome is a paradoxical response to drainage of a pericardial effusion resulting in cardiovascular collapse. This syndrome carries a high mortality rate and thus should be recognized and managed immediately. Although often associated with left ventricular failure, isolated right ventricular failure can also occur. Management should be targeted towards optimizing right heart function via inotropic support and maintaining euvolemia. In severe cases, emergent cannulation and initiation of cardiopulmonary bypass may be required. This syndrome is transient, with most patients fully recovering systolic function. Thus, a cardiopulmonary bypass in the operating room may provide the best setting for allowing cardiac recovery. Other forms of cardiopulmonary bypasses, such as VA-ECMO or other ventricular support devices, can be considered when systolic failure persists for longer.
